# Cardioprotective Effect of *Rheum turkestanicum* Against Doxorubicin-Induced Toxicity in Rats

**DOI:** 10.3389/fphar.2022.909079

**Published:** 2022-06-08

**Authors:** Azar Hosseini, Mohammad-Kazem Safari, Arezoo Rajabian, Samaneh Boroumand-Noughabi, Ali H. Eid, Yusra Al Dhaheri, Eric Gumpricht, Amirhossein Sahebkar

**Affiliations:** ^1^ Department of Pharmacology, Faculty of Medicine, Mashhad University of Medical Sciences, Mashhad, Iran; ^2^ Pharmacological Research Center of Medicinal Plants, Mashhad University of Medical Sciences, Mashhad, Iran; ^3^ Department of Internal Medicine, Faculty of Medicine, Mashhad University of Medical Sciences, Mashhad, Iran; ^4^ Department of Pathology, Faculty of Medicine, Mashhad University of Medical Sciences, Mashhad, Iran; ^5^ Department of Basic Medical Sciences, College of Medicine, QU Health, Qatar University, Doha, Qatar; ^6^ Department of Biology, College of Science, United Arab Emirates University, Al-Ain, United Arab Emirates; ^7^ Isagenix International LLC, Gilbert, AZ, United States; ^8^ Biotechnology Research Center, Pharmaceutical Technology Institute, Mashhad University of Medical Sciences, Mashhad, Iran; ^9^ Applied Biomedical Research Center, Mashhad University of Medical Sciences, Mashhad, Iran; ^10^ School of Medicine, The University of Western Australia, Perth, WA, Australia; ^11^ Department of Biotechnology, School of Medicine, Mashhad University of Medical Sciences, Mashhad, Iran

**Keywords:** *Rheum turkestanicum*, chemotherapy, doxorubicin, oxidative stress, herbal medicine, cardiotoxicity

## Abstract

**Background:** Doxorubicin as an anti-cancer drug causes cardiotoxicity, limiting its tolerability and use. The mechanism of toxicity is due to free radical production and cardiomyocytes injury. This research evaluated *Rheum turkestanicum* (*R.turkestanicum*) extract against doxorubicin cardiotoxicity due to its considerable *in vitro* antioxidant activity.

**Methods:** Male Wistar rats received 2.5 mg/kg doxorubicin intraperitoneally every other day for 2 weeks to create an accumulative dose. *R. turkestanicum* was administrated at a dose of 100 and 300 mg/kg intraperitoneally from the second week for 7 days. On the 15th day, the animals were anesthetized and blood was collected from cardiac tissue for evaluation of alanine aminotransferase (ALT), cardiac muscle creatinine kinase (CK-MB), troponin T (cTn-T), lactate dehydrogenase (LDH), and B-type natriuretic peptide brain natriuretic peptide. A cardiac homogenate was also collected to determine superoxide dismutase (SOD), catalase Catalase Activity, malondialdehyde (MDA), and thiols. Histopathology was also performed.

**Results:** Doxorubicin increased all cardiac enzymes and malondialdehyde, correlating with a reduction in SOD, catalase, and thiols. Histopathology revealed extracellular edema, moderate congestion, and hemorrhage of foci. In contrast, administration of *R. turkestanicum* ameliorated these doxorubicin-induced pathophysiological changes.

**Conclusion:** This study revealed that the extract ameliorated doxorubicin-induced cardiac toxicity via modulation of oxidative stress-related pathways. Liquid chromatography-mass spectrometry analysis of *R. turkestanicum* indicated several components with potent pharmacological properties.

## Introduction

Doxorubicin is a commonly administered chemotherapeutic agent for a variety of cancers. However, doxorubicin’s utility is restricted due to its associated cardiotoxicity, with a reported incidence of toxicity of approximately 11% (Chatterjee et al., 2010). Additionally, another study showed that 2.2% of 4,000 patients who consumed doxorubicin had symptoms of heart failure (Bennink et al., 2004). This cardiotoxicity involves metabolic activation to a semiquinone, subsequent oxidative stress, and binding of lipid peroxidation products [such as malondialdehyde (MDA)] toward macromolecular targets (Ma et al., 2013). The administration of doxorubicin also attenuates antioxidant enzymes such as catalase (CAT), glutathione peroxidase (GSH), and superoxide dismutase (SOD) (Kosoko et al., 2017). Moreover, doxorubicin stimulates apoptosis in cardiomyocytes resulting in congestive heart failure. Clinically, heart failure is associated with symptoms such as edema, orthopnea, fatigue, and increased venous pressure, which promote cardiac dysfunction (Yancy et al., 20132013). Following heart failure, the level of biomarkers such as lactate dehydrogenase (LDH), creatinine kinase (CK-MB), troponin T, and *brain natriuretic peptide* (*BNP*) elevate rapidly (Liquori et al., 2014). Therefore, researchers seek additional protective agents to mitigate doxorubicin’s cardiotoxicity. One such agent, dexrazoxane, can reduce doxorubicin toxicity; however, this drug interacts with the chemotherapeutic benefits of doxorubicin (Swain and Vici, 2004). Researchers are now investigating other natural compounds with lower side effects, including herbal medicines (Hosseini and Sahebkar, 2017). Phytochemical-rich products are candidates for reducing the cardiotoxicity of doxorubicin because of their antioxidant and cardioprotective properties (Hosseini and Sahebkar, 2017). *Rheum turkestanicum (R.turkestanicum)* belongs to the polygonaceae family. It grows in Asia, especially in the northeast of Iran (Taheri and Assadi, 2013). The studies have reported that *R. turkestanicum* has beneficial effects in treating diabetes, hypertension, and cancer (Shiezadeh et al., 2013; Moradi et al., 2016; Boroushaki et al., 2019a; Moradzadeh, 2019; Ghorbani et al., 2021). This herb is composed of different chemical components, including anthraquinones (e.g., aloe-emodin, emodin glycosides, physione, and rhein), flavonoids (e.g., epicatechin and quercetin), alkanes (e.g., eicosane and heneicosane), and fatty acids (e.g., linoleic acid and 9-octadecenoic acid) (Hosseini et al., 2017). Therefore, the pharmacological properties of *R. turkestanicum* can be related to the presence of active ingredients (Ghorbani et al., 2019). One recent study showed *R. turkestanicum* reduced doxorubicin-induced toxicity in cardiomyocytes (H9c2 cell line) via modulation of oxidative stress (Hosseini and Rajabian, 2016). Also In another study cardioprotective effect of *R. turkestanicum* against isoprenaline was evaluated which showed anti-oxidant activity of extract reduced isoprenaline-induced cardiac toxicity (Hossini et al., 2022) In the current study, we have evaluated the cardioprotective effects of *R. turkestanicum* against doxorubicin-induced cardiotoxicity in an animal model.

## Materials and Methods

### Reagents

Doxorubicin, ketamine hydrochloride/xylazine hydrochloride solution and 2-thiobarbituric acid (TBA) were prepared from Sigma-Aldrich (St. Louis, MO, United States). Pyrogallol and 2,2′ -dinitro-5,5′ -dithiodibenzoic acid (DTNB) were obtained from Cayman (Michigan, United States). Other agents included alanine aminotransferase (ALT) (mancompany, 613,032), muscle, brain creatinine kinase (CK-MB) (Riton, R144), troponin T (Biomerieux, 415,386), lactate dehydrogenase (LDH) (mancompany, 613,036), and B-type natriuretic peptide (BNP) (Sigma Aldrich, RAB0386). CK-MB was measured by VIDAS device according to enzyme-linked fluorescent assay and other biochemical tests were carried out by autoanalyzer Hitachi 902.

### Preparation of *R. turkestanicum* Extract

*R. turkestanicum* was collected from chenar trees around Khorasan and identified by a botanist. The voucher specimen is 21,377. The root of *R. turkestanicum* was dried and converted to a fine powder. The hydro-alcoholic extract was obtained by the Soxhlet apparatus. After 48 h, the extract was dried in a water bath and maintained at −18°C until use. The final yield of the extract was determined as 21% (w/w).

### Animals

This project was done according to the National Institutes of Health (NIH) Guide for Laboratory Animals and was confirmed by the Animal Ethics Committee of Mashhad University of Medical Sciences, Mashhad, Iran (IR.MUMS.MEDICAL.REC.1399.425). Adult male albino Wistar rats (200–250 g) were housed at the Laboratory Animals Research Center, Mashhad University of Medical Sciences, at 22 ± 4°C with a 12 h dark/light cycle. The animals had free access to standard laboratory chow and tap water *ad libitum*.

### Experimental Protocol

The rats were divided into five groups, with eight rats in each group. Group 1 received normal saline 0.9% intraperitoneally (i.p.) for 14 days. Doxorubicin was administrated i. p. at a dose of 2.5 mg/kg in 6 equal injections for 2 weeks to create a cumulative dose (15 mg/kg body weight) (Arafa et al., 2014). Groups 3 and 4 received *R. turkestanicum* at doses of 100 and 300 mg/kg (Hosseini et al., 2017; Jahani Yazdi et al., 2020; Hossini et al., 2022) as i. p. one week after doxorubicin administration. *R. turkestanicum* was injected at a dose of 300 mg/kg to rats in the fifth group without administration of doxorubicin. On the 15th day, ketamine was injected at 75 mg/kg i. p to induce anesthesia. Three ml of blood was collected from the heart, then centrifuged for 10 min at 1000 rpm. The separated serums were kept at −20°C to determine biochemical parameters such as alanine aminotransferase (ALT) (mancompany, 613,032), muscle, and brain creatinine kinase (CK-MB) (Riton, R144), Troponin T (Biomerieux, 415,386), lactate dehydrogenase (LDH), and B-type natriuretic peptide (BNP) (Sigma Aldrich, RAB0386). The isolated cardiac tissue was homogenized in cold KCl solution (1.5%, pH = 7) to give a 10% homogenate, and used for suspension to measure thiol, malondialdehyde (MDA), an anti-oxidant enzyme levels.

### Liquid Chromatography-Mass Spectrometry Analysis of *R. turkestanicum*

According to our previous study, the LC-MS analysis was performed using an AB SCIEX QTRAP (Shimadzu) liquid chromatography coupled with a triple quadrupole mass spectrometer (Hossini et al., 2022). Liquid chromatography separation was performed on a Supelco C18 (15 mm × 2.1 mm × 3 μm) column. MS analysis was carried out in negative and positive ionization modes to monitor as many ions as possible and ensure that the most significant number of metabolites extracted from the sample was detected. The analysis was done at a flow rate of 0.5 ml/min. The gradient analysis started with 95% of 0.2% aqueous formic acid, isocratic conditions were maintained for 1 min, and then a 14-min linear gradient to 40% acetonitrile with 0.2% formic acid was applied. From 15 to 35 min, the acidified acetonitrile was increased to 100%, followed by 5 min of 100% acidified acetonitrile and 5 min at the start conditions to re-equilibrate the column. The mass spectra were acquired in a range of 150–1,000 within 45 min of scan time. Mass feature extraction of the acquired LC-MS data and maximum detection of peaks were done using the MZmine analysis software package, version 2.3.

### Lipid Peroxidation Analysis

Lipid peroxidation was assessed *via* MDA generation as previously reported (Boroushaki et al., 2019a). The samples were mixed with thiobarbituric acid (TBA) (0.67%) and trichloroacetic acid (10%), then boiled for 40 min. HCl and n-butanol were added to cooled samples, centrifuged, and the upper layer was measured spectrophotometrically at 535 nm. MDA concentration (M) was determined as: Absorbance/(1.56*105 cm^−1^M^−1^).

### Determination of Thiols

Thiol concentrations were determined using DTNB as previously described (11). Briefly, Tris-EDTA buffer (pH = 8.6) was added to 50 µl of the homogenate, then the absorbance was read at 412 nm (A1). The solution was mixed with DTNB and again measured after 15 min (A2). DTNB was applied as blank. Total thiol concentration (mM) = (A2-A1-B)*0.7)/0.05*14.

### Determination of Enzyme Markers and Biochemical Parameters

The level of markers such as LDH, CK-MB, cTnT, BNP and ALT was determined according to standard kits and manufacturer’s instructions.

### Determination of Catalase Activity (CAT)

Catalase activity was determined according to (Aebi, 1984). This protocol’s based on the constant rate (k) (dimension: s-1, k) of hydrogen peroxide reduction measuring absorbance at 240 nm. The activity of CAT was expressed as K (rate constant) per liter.

### Evaluation of Superoxide Dismutase (SOD) Activity

Superoxide dismutase activity was determined colorimetrically according to (20). This procedure measures superoxide production by pyrogallol auto-oxidation, and the prevention of superoxide-dependent reduction of the tetrazolium dye to its Formosan by SOD was evaluated at 570 nm (Madesh and Balasubramanian, 1998).

### Histopathological Analysis

The isolated hearts were fixed with 10% formalin solution for histopathological studies. After paraffinization of tissues, the slices of 3 mm thickness were prepared. Hematoxylin and Eosin were used to dye the sections for light microscopic analysis.

### Statistical Analyses

Data were analyzed using GraphPad Prism^®^ software version 8 (GraphPad Software, San Diego, CA) and presented as means ± SD. Significance was determined by One-Way ANOVA followed by the Tukey-Kramer test.

## Results

### LC-MS Analysis of *R. turkestanicum* Extract

**FIGURE 1:**
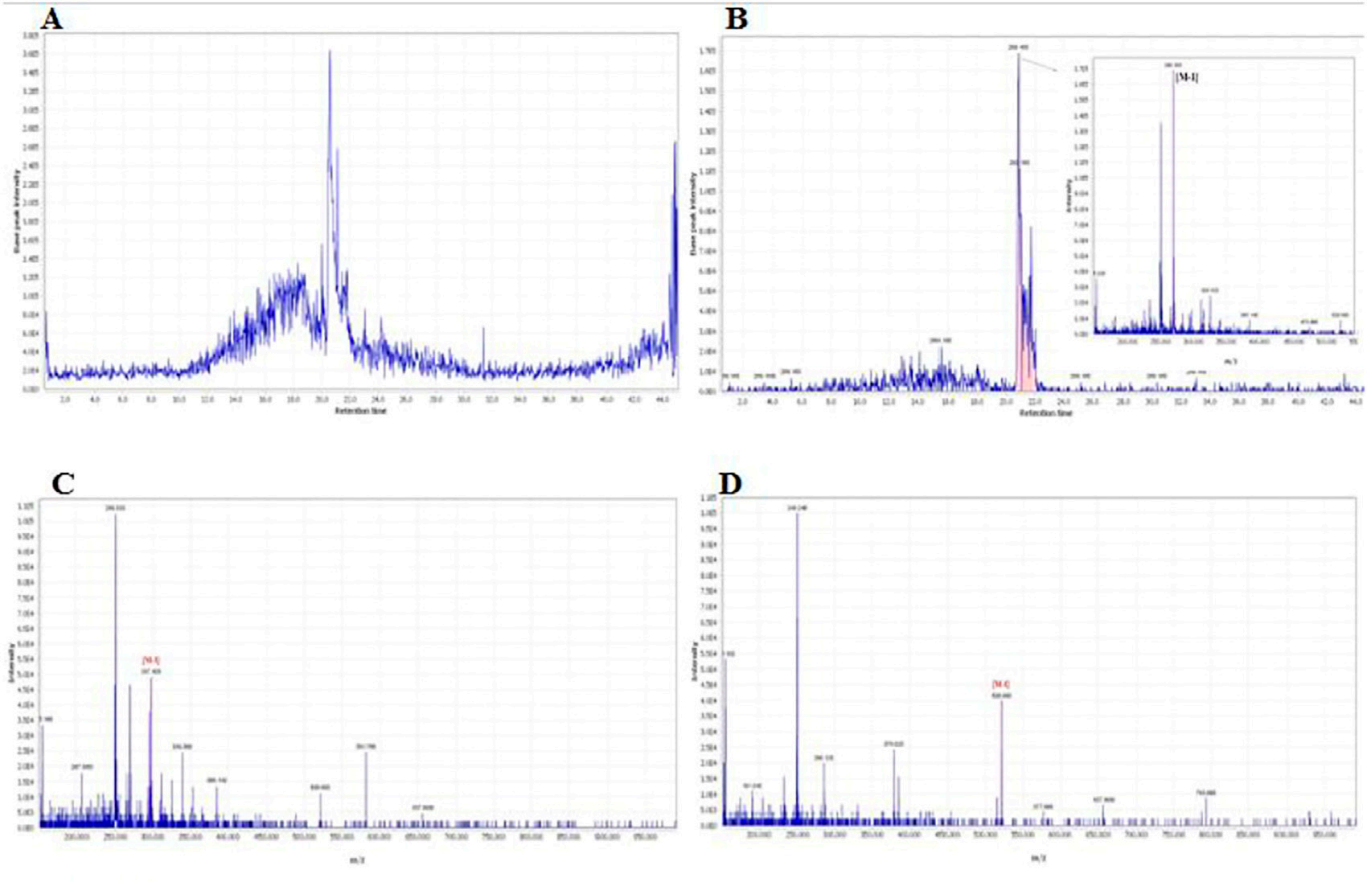
Chromatogram and corresponding mass adducts which was reported in our recent work (Hossini et al., 2022). **(A)** The total ion chromatogram of *R.turkestanicum* using LC-MS in the positive mode. **(B)** Chromatogram of emodin and corresponding mass adduct, [M-H], at m/z 269.16. **(C)** Mass spectra of 6-methyl-rhein, [M-H], at m/z 297.42. **(D)** Mass spectra of revandchinone 1, [M-H], at m/z 520.98.

A total of 24 compounds were identified in the hydro-ethanol extract of *R. turkestanicum* using LC-MS analysis. These compounds include anthraquinones (e.g., emodin, emodin glycosides, physione, rhein, and its derivatives), fatty acids (9-octadecenoic acid), and flavonoids (e.g., epicatechin and quercetin). The extract also contained a high level of glucogallin, a phenolic compound formed from β-D-glucose and gallic acid (Hosseini et al., 2017). Identification of the compounds are shown in Table 1. The total ion chromatograms of *R. turkestanicum* extract in ESI− mode are shown in Figure 1. The MS spectral data were compared with the reported compounds in some previous literature. Figures 1A–D provide a representative chromatogram.

**TABLE 1:** Peak assignment of metabolites in the hydro-ethanol extract of R.turkestanicum using LC-MS in the negative mode (Hossini et al., 2022).

Peak no.	Compound	RT (min)	[M-1] (*m/z*)	Intensity (E)	References
1	6-methyl-rhein	21.3	297.42	4.94	Zargar et al. (2011)
2	6-methyl-rhein-diacetate	31.8	381.06	2.74	Singh et al. (2005)
3	Emodin	20.9	269.16	1.75	Zhang and Liu (2004), Zargar et al. (2011)
4	Emodin-8-O-glucopyranoside	18.0	431.28	2.24	Ko et al. (1995), Matsuda et al. (2000)
5	Emodin glucoside	18.2	431.34	1.64	Zargar et al. (2011)
6	Revandchinone 1	17.1	520.98	4.04	Zargar et al. (2011)
7	Revandchinone 2	20.0	674.58	3.84	Zargar et al. (2011)
8	Revandchinone 3	2.5	577.68	2.44	Zargar et al. (2011)
9	Chrysophanol	20.5	254.94	2.74	Zhang and Liu (2004), Zargar et al. (2011)
10	Epicatechin	44.4	289.08	2.04	Zargar et al. (2011)
11	Ethyl linoleate	44.2	307.62	3.84	Ragasa, (2017)
12	Glucogallin	32.0	331.08	2.24	Thakur et al. (1989)
13	Danthron	37.7	239.22	2.24	Chen et al. (2019)
14	Methyleugenol	19.1	177.3	9.14	Miyazawa et al. (1996)
15	Physcion	19.6	283.44	2.74	Zhang and Liu (2004), Zargar et al. (2011)
16	Piceatannol	23.1	243.36	8.74	Raalh and Tsouprasd, (2003)
17	Epigallocatechol	44.5	305.04	6.74	Agarwal et al. (2001)
18	Cadinen	10.9	204.36	3.14	Miyazawa et al. (1996)
19	9-octadecenoic acid	30.2	281.10	2.04	Ragasa, (2017)
20	Quercetin	44.9	300.60	3.64	Ragasa, (2017)
21	Rhaponticin-β-D-glucoside	21.1	717.00	2.65	Ragasa, (2017)
22	Rhein	19.3	283.38	2.44	Zhang and Liu (2004), Zargar et al. (2011)
23	Rheochrysin	20.4	444.78	6.24	Youngken (1946), Agarwal et al. (2001)
24	Rhododendrin	19.9	327.36	2.14	Dehghan et al. (2018)

### Effect of *R.turkestanicum* on Cardiac Parameters

**FIGURE 2:**
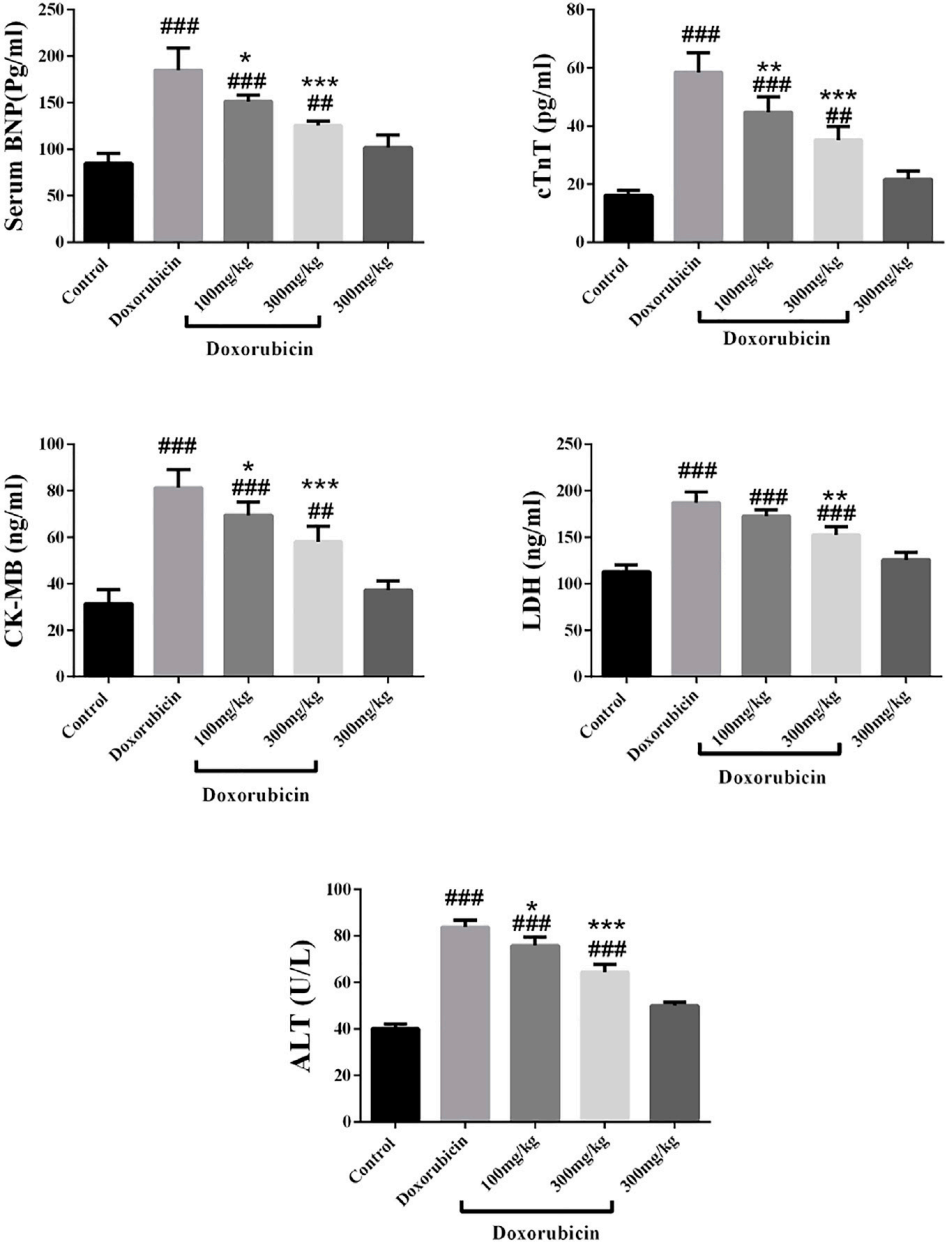
Effect of *R. turkestanicum* on the level of serum BNP, cTnT, CK-MB, LDH and ALT. Data were expressed as mean ± SD. ##*p <* 0.01, ###*p <* 0.001 in comparison with the control group. *p < 0.05, **p < 0.01, ***p < 0.001 in comparison with doxorubicin.

**FIGURE 3:**
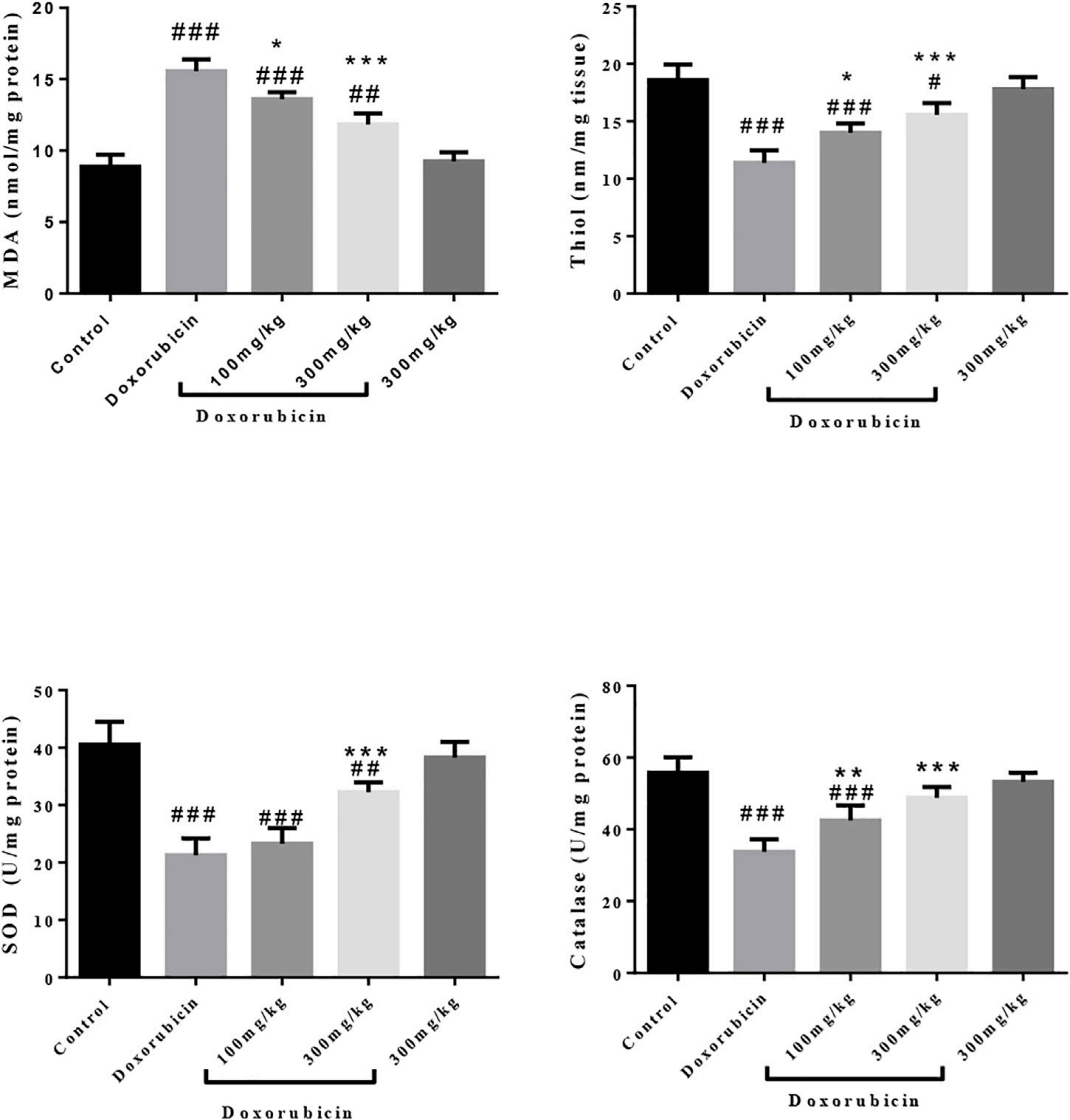
Effect of *R. turkestanicum* on oxidative stress and antioxidant enzymes. Data were expressed as mean ± SD. #*p <* 0.05, ##*p <* 0.01, ###*p <* 0.001 in comparison with the control group. *p < 0.05, **p < 0.01, and ***p < 0.001 in comparison with doxorubicin.

Our results revealed that doxorubicin increased the level of BNP, CK-MB, cTnT, LDH and ALT significantly in comparison with the control group (*p* < 0.001) (Figure 3 ). At the dose of 100 mg/kg, *R. turkestanicum* significantly reduced levels of BNP (*p* < 0.05), CK-MB (*p* < 0.05), cTn-T (*p* < 0.01), and ALT (*p* < 0.05). The higher dose of the extract (300 mg/kg) further abrogated doxorubicin-induced elevations of marker enzymes, including BNP, cTn-T, CK-MB and ALT (*p* < 0.001), and LDH (*p* < 0.01) in comparison with doxorubicin alone (Figure 2). *R. turkestanicum* alone had no effect on any of these cardiac enzymes.

### Effect of *R.turkestanicum* on Oxidative Stress

As shown in Figure 3, overall, doxorubicin increased oxidative stress while simultaneously reducing antioxidant defenses. Specifically, the cardiotoxic drug increased MDA production (*p* < 0.001) and reduced thiols (*p* < 0.001), and enzymatic activities of SOD and catalase (*p* < 0.001) compared to control. Administration of R. turkestanicum ameliorated doxorubicin-induced changes. At dosages of both 100 mg/kg and 300 mg/kg, the extract prevented oxidative stress (*p* < 0.05 and *p* < 0.001, respectively) and restored thiol content (*p* < 0.05 and *p* < 0.001, respectively). Similarly, both dosages of R. turkestanicum significantly increased both SOD (*p* < 0.001 at 300 mg/kg) and catalase (*p* < 0.01 and *p* < 0.001 at 100 mg/kg and 300 mg/kg, respectively). The extract alone had no effect on either biomarkers of oxidative stress and antioxidant defenses.

### Histopathological Analysis

**FIGURE 4:**
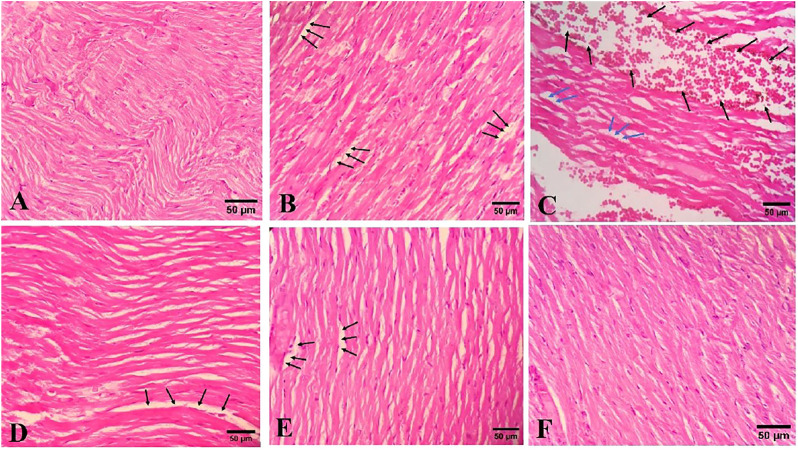
The effects of *R. turkestanicum* on cardiac histopathology. **(A)** (H&E, ×400) Control group shows normal cardiac tissue, **(B)** (H&E ×400) doxorubicin group shows edema (black arrows) and **(C)** (H&E ×400) doxorubicin group indicates edema (blue arrows) and hemorrhage (black arrows), **(D)** (H&E ×400) *R.turkestanicum* + doxorubicin (100 mg/kg) shows mild edema (black arrows), **(E)** (H&E ×400) *R.turkestanicum* + doxorubicin (300 mg/kg) also exhibits mild edema (black arrows), and **(F)** (H&E ×400) *R.turkestanicum* (300 mg/kg) shows no obvious pathological changes and is similar to control group.

Microscopic examination of the doxorubicin-treated group showed mild to moderate degrees of mostly extracellular edema and moderate congestion and small foci of hemorrhage (Figure 4). The tissue changes in groups 3 and 4 (doxorubicin + extract), included mild edema and mild congestion. These changes were similar but lessened at the 300 mg/kg dose. Again, *R. turkestanicum* alone had no effect on histopathology.

## Discussion

Although doxorubicin is a drug of choice for various cancer treatments, its cardiotoxicity restricts its clinical utility. The primary finding of this study is that *R. turkestanicum* reduced doxorubicin-induced cardiotoxicity by modulating oxidative stress and antioxidant defenses. The extract also reduced the drug’s effect on pathological abnormalities.

Several mechanisms may be responsible for doxorubicin-induced cardiotoxicity. These mechanisms include mitochondrial injury, ROS generation, intracellular Ca+2 dysregulation, inflammatory cytokine production, and myocyte damage (Rawat et al., 2021; Sheibani et al., 2022; Wu et al., 2022).

Doxorubicin is metabolized *via* several oxidative/reductive enzymes into a semiquinone, which increases the generation of superoxide radicals via redox cycling. Relatedly, doxorubicin facilitates oxidative stress by impairing antioxidant defense enzymes such as SOD and catalase. Concomitantly, this oxidative stress is associated with a depletion of glutathione (GSH), the primary thiol antioxidant within cells. Reduced GSH levels are also important in the doxorubicin-induced downregulation of GSH-Px 4 (Tadokoro et al., 2020). Restoration of GSH status protects against the drug’s cardiotoxicity (Mohamed et al., 2000). Interestingly, Hosseini *et al.* reported that *R. turkestanicum* prevented doxorubicin-induced cardiotoxicity, similar to intervention with N-acetylcysteine, a structurally related non-protein thiol antioxidant (Hosseini and Rajabian, 2016). Maintenance of GSH-dependent antioxidant defenses and various other compounds, including carvedilol, omega-3 fatty acids, and dexrazoxane, attenuate some of the drugs’ toxicity (Takemura and Fujiwara, 2007).

Doxorubicin preferentially accumulates in the cardiac mitochondria and is associated with cardiac toxicity. For example, doxorubicin significantly reduces mitochondrial complex 1 activity and can promote apoptosis (Marcillat et al., 1989; Basit et al., 2017; Rawat et al., 2021). Additionally, the expression of PARP1 in cardiomyocytes impairs mitochondrial function (Wen et al., 2018). Therefore, stabilizing the mitochondria can prevent doxorubicin-induced cardiotoxicity (Montaigne et al., 2010). Indeed, recent studies have reported that natural products may be appropriate alternatives for chemical agents in various diseases (Momtazi et al., 2017; Bagherniya et al., 2018; Alidadi et al., 2020; Soltani et al., 2021). One such natural product, *R. turkestanicum*, belongs to the Polygonaceae family and is composed of active ingredients with various pharmacological and biochemical properties (Ghorbani et al., 2019). The current study supports an earlier report that *R. turkestanicum* can reduce doxorubicin-induced toxicity in myocytes (Hosseini and Rajabian, 2016). In that study, the extract’s protective effects against doxorubicin were evaluated *in vivo*. Our findings confirm doxorubicin-induced cardiotoxicity via mechanisms involving elevated oxidative stress and reduced antioxidant defenses (Songbo et al., 2019). Other studies have found that *R. turkestanicum* could decrease oxidative stress in endothelial cells (Hosseini et al., 2020).

Patients receiving doxorubicin exhibit left ventricular dysfunction demonstrated by elevated CK-MB and BNP activities (Pongprot et al., 2012). Additionally, animal studies have reported increased CK-MB, LDH, BNP, and cTnT activities following doxorubicin administration (Arafa et al., 2014; Kulkarni and Swamy, 2015; Nugraha et al., 2020; Syahputra et al., 2021). Moreover, previous research has also reported that doxorubicin elevates ALT (Barakat et al., 2018); our findings support these previous ones of doxorubicin-induced clinical enzyme alterations. Our findings also support that *R. turkestanicum* protects against doxorubicin-induced cardiotoxicity by restoring cellular antioxidant defenses and as an antioxidant in various cells and toxicity models (Hosseini et al., 2018; Boroushaki et al., 2019a; Boroushaki et al., 2019b; Hosseini et al., 2020). The protection by the plant is also associated with improvements in LDH and creatinine phosphokinase activities (Hosseini et al., 2017; Hossini et al., 2022). The protective effect of *R. turkestanicum* observed in our study is potentially attributed to the presence of specific plant bioactives. Specifically, *R. turkestanicum* is rich in molecules offering cellular and tissue protection, including emodin, rhein, epicatechin, and others, as we show in Table 1 and as reported by others Dehghan et al., 2018. As one example of these bioactives, it was reported that chrysophanol could improve cardiac function via inhibition of apoptosis, modulation of oxidative stress, and prevention of c-Jun N-terminal kinase (JNK 1/2) activation (Xie and Li, 2020). Additionally, chrysophanol reduced doxorubicin-induced cardiotoxicity by suppressing mitochondrial swelling, mitochondrial depolarization, and PARP1 inhibition (Lu et al., 2019). Epicatechin reduces blood pressure in hypertensive patients and infarct size (Yamazaki et al., 2008; Yamazaki et al., 2010). Another bioactive in *R. turkestanicum*, rhein, had demonstrated cytoprotection against oxidative stress-induced endothelial cell injury (Zhong et al., 2012). Finally, the cardioprotective effects of emodin and quercetin have been reported (Wu et al., 2007; Dong et al., 2014) and include the potential modulation by emodin of doxorubicin-induced cardiotoxicity *via* reduced Toll-like receptor four and P38 mitogen activating protein kinase (P38 MAPK) expression (Zhang et al., 2016).

## Conclusion

The protective effect of *R. turkestanicum* against doxorubicin-induced cardiotoxicity is attributed, at least in part, to the plant’s antioxidant properties and enhancement of cardiac tissue antioxidant defenses. However, our study has some limitations, such as the lack of cardiac function evaluation by echocardiography and the unknown mechanistic effects of co-administration of *R. turkestanicum* and doxorubicin. These considerations will be necessary in future studies. Future studies are also warranted to test the impact of extract upon oral administration, and perform quantitative analysis of *R. turkestanicum* extract and identify the main active phytochemicals responsible for the plant’s cardioprotection.

## Data Availability

The raw data supporting the conclusion of this article will be made available by the authors, upon a reasonable request.
